# Tibial Plateau Fracture with Avulsion Fracture of Tibial Tuberosity: A Case Report and Review of Literature

**DOI:** 10.7759/cureus.7756

**Published:** 2020-04-21

**Authors:** Rajesh Rana, A. Ganesh, Sudarsan Behera, Himansu Behera

**Affiliations:** 1 Orthopaedics, All India Institute of Medical Sciences, Bhubaneswar, IND; 2 Orthopaedics, Institute of Medical Sciences and SUM Hospital, Bhubaneswar, IND; 3 Orthopaedics, All India Institute of Medical Sciences, Bhubaneshwar, IND

**Keywords:** tibial tuberosity fracture, tibial plateau fracture, rare, internal fixation

## Abstract

Tibial tuberosity fractures are usually seen in the adolescent age group and are very rare in adults. Here we describe a case of knee injury following a road traﬃc accident. The patient had tibial tuberosity avulsion along with tibial plateau fracture. He was treated with open surgical reduction and internal ﬁxation. His rehabilitation was quite successful, resulting in a good range of motion and functional outcome. This study aims to present this unusual case of tibial tuberosity avulsion fracture associated with tibial plateau fracture and its appropriate management.

## Introduction

Tibial tuberosity fractures are rare and even rarer in adults [1]. A tibial tuberosity fracture associated with a tibial plateau fracture is rare. The incidence of tibial tuberosity fracture is only 3% among proximal tibia fractures [2,3]. It is more common in the adolescent age groups when the muscle, ligament, and tendons are stronger than bone [4]. In adolescent groups, this fracture is mainly due to jumping and tackling activities [5]. Tibial plateau fractures result from high-energy trauma and can involve the tibial tubercle, which represents a disruption in the extensor mechanism and, logically, must be stabilized. To date, very few cases of tibial plateau fracture with tibial tuberosity avulsion have been reported. Here we present a case with Schatzker type VI fracture with a tibial tuberosity avulsion fracture. According to the Arbeitsgemeinschaft für Osteosynthesefragen (AO) classiﬁcation, our tibial plateau fracture is type 41C1 and the tibial tuberosity fracture is type 41A1.2.

## Case presentation

**Figure 1:**
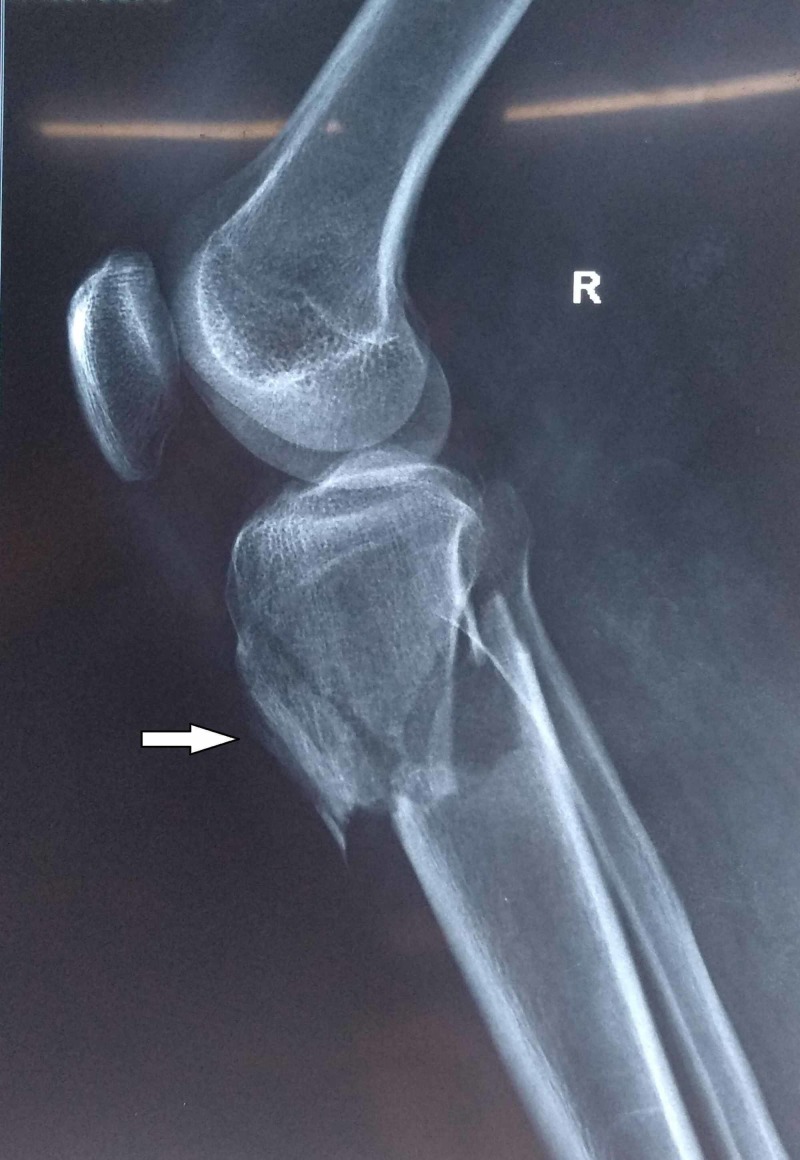
Tibial plateau fracture with tibial tuberosity avulsion fracture radiograph (white arrow)

A 31-year-old male patient came to the hospital following a road traﬃc accident with pain in the right knee and unable to walk. On examination, there was swelling and tenderness, and redness was present over the right knee and the proximal part of the leg. There was no distal neurovascular deﬁcit with dorsalis pedis, tibialis posterior pulsations were well felt, and the sensation was intact. Compartment syndrome was ruled out as there were no signs. The radiograph of the knee was taken in both anteroposterior and lateral views. The radiograph showed tibial plateau fracture with tibial tuberosity fracture (Figure 1), with the tibial plateau fracture extending up to metaphysis.

**Figure 2:**
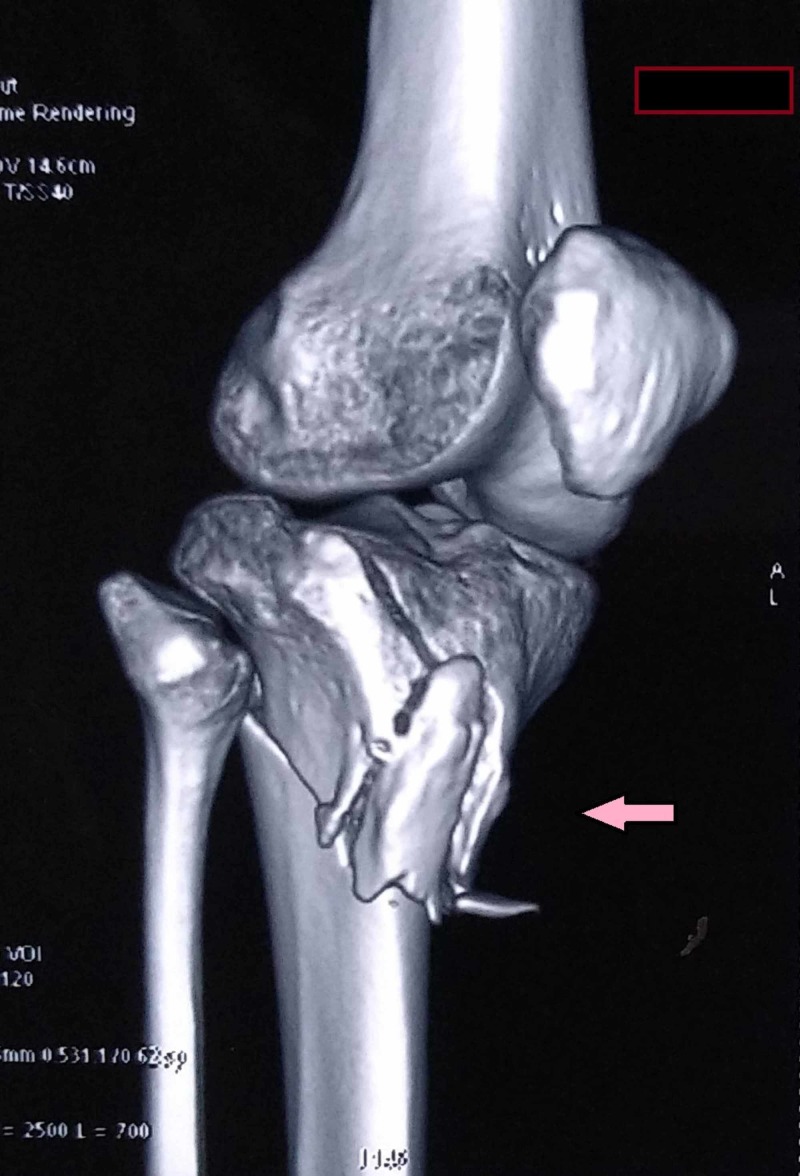
Three-dimensional computed tomography image showing a bicondylar fracture with tuberosity fracture (pink arrow)

A three-dimensional computed tomography (CT) scan of the knee and proximal tibia was done. The CT scan showed a bicondylar fracture with a metaphyseal extension of fracture and tuberosity fracture (Figure 2).

**Figure 3:**
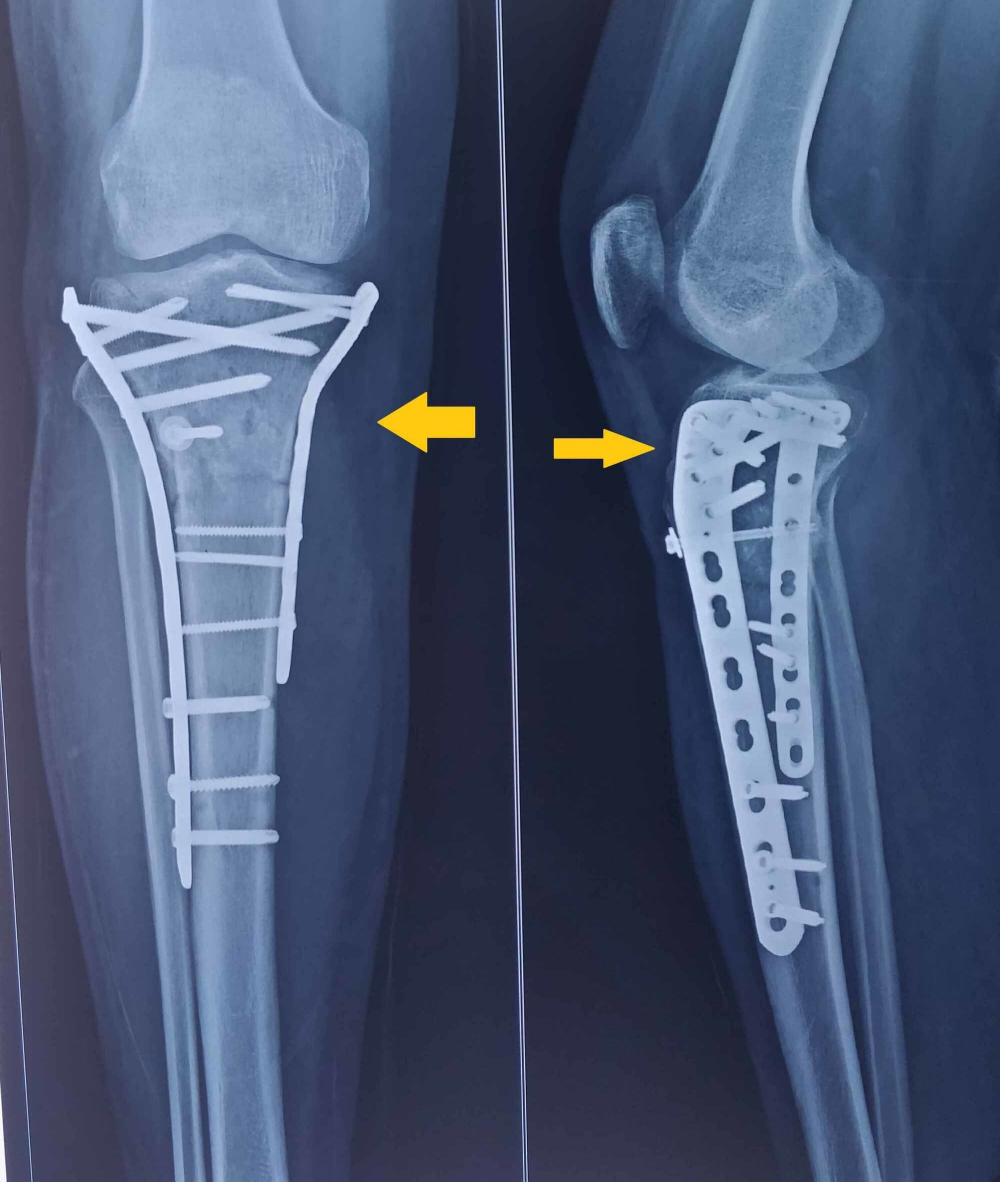
Postoperative radiograph of the knee showing tibial tuberosity avulsion fracture, which was reduced and ﬁxed with an anteroposterior screw with a washer (yellow arrows)

According to AO classiﬁcation, the tibial plateau fracture was a complete articular type with 41C1 and tibial tuberosity fracture. Open reduction and internal ﬁxation were planned. First, by the posteromedial approach, a posteromedial buttress plate was fixed for the posteromedial fragment. Then the lateral spilt depression fracture was reduced and ﬁxed with a hockey stick locking plate. The tibial tuberosity avulsion fracture was reduced and ﬁxed with an anteroposterior screw with a washer (Figure 3).

All fractures and the articular surface were reduced and ﬁxed under ﬂuoroscopy guidance. After one week, the patient was allowed to bend his knee and exercise his quadriceps. After six weeks, he was allowed to walk with a walker and partially bear weight on his right leg. After four months, he was allowed to bear full weight. The patient resumed normal activity after six months with no restriction of movements.

## Discussion

A tibial tuberosity avulsion fracture is common in the adolescent age group, but is rare in adults. It is more common in men and most commonly occurs with a sudden contracture of the quadriceps muscle [6]. It is commonly associated with jumping and sudden knee ﬂexion activities. Very few cases of tibial plateau fractures have been reported. There is little literature showing a fracture of the bicondylar proximal tibia with tibial tuberosity fracture. There is a risk of missing a tibial tuberosity fracture when observing the main fracture fragments for ﬁxation. This may result in painful rehabilitation and may lead to nonunion of the tibial tuberosity resulting in extensor lag. Tibial tuberosity should be ﬁxed separately, and preferably with a screw, because there is very little soft tissue overlying it [7]. The prognosis is generally good if it is diagnosed properly and surgically ﬁxed. There is, however, an increased chance of re-rupture and stiffness following tibial tuberosity fracture.

## Conclusions

Tibial tuberosity fracture along with tibial plateau fracture is less common, and care should be taken to ensure it is not missed. It is even rarer in the adult population. Tibial tuberosity avulsion is missed many a times. Proper imaging including CT scan should be done. Tibial plateau fracture should be fixed with appropriate internal fixation and simultaneously tibial tuberosity avulsion should be addressed. Early rehabilitation should be started to prevent stiffness. With proper surgical ﬁxation and early rehabilitation, a good functional outcome can be achieved.
